# Bioinspired mechanically active adhesive dressings to accelerate wound closure

**DOI:** 10.1126/sciadv.aaw3963

**Published:** 2019-07-24

**Authors:** S. O. Blacklow, J. Li, B. R. Freedman, M. Zeidi, C. Chen, D. J. Mooney

**Affiliations:** 1 John A. Paulson School of Engineering and Applied Sciences, Harvard University, Cambridge, MA 02138, USA.; 2 Wyss Institute for Biologically Inspired Engineering, Harvard University, Cambridge, MA 02138, USA.; 3 School of Medicine, University of California, San Francisco, San Francisco, CA 94143, USA.; 4 Department of Bioengineering, University of California, Berkeley, Berkeley, CA 94720, USA.; 5 Department of Mechanical Engineering, McGill University, Montreal, QC H3A 0G4, Canada.; 6 Department of Biomedical Engineering, McGill University, Montreal, QC H3A 0G4, Canada.

## Abstract

Inspired by embryonic wound closure, we present mechanically active dressings to accelerate wound healing. Conventional dressings passively aid healing by maintaining moisture at wound sites. Recent developments have focused on drug and cell delivery to drive a healing process, but these methods are often complicated by drug side effects, sophisticated fabrication, and high cost. Here, we present novel active adhesive dressings consisting of thermoresponsive tough adhesive hydrogels that combine high stretchability, toughness, tissue adhesion, and antimicrobial function. They adhere strongly to the skin and actively contract wounds, in response to exposure to the skin temperature. In vitro and in vivo studies demonstrate their efficacy in accelerating and supporting skin wound healing. Finite element models validate and refine the wound contraction process enabled by these active adhesive dressings. This mechanobiological approach opens new avenues for wound management and may find broad utility in applications ranging from regenerative medicine to soft robotics.

## Teaser

Bioinspired adhesive dressings have been shown to shrink at skin temperature, contract the wound, and promote wound healing.

## INTRODUCTION

Wound management remains a central concern in clinical care because of the constant incidence of traumatic injuries, the rising prevalence of chronic wounds such as diabetic ulcers and pressure sores, and aging populations that exhibit diminished wound healing ability (*1*–*3*). Skin injuries are painful and can compromise the integrity and protective function of the skin, resulting in infections. Current treatment options involve gauzes, cotton wools, and dressings, which aim to primarily maintain the moisture at wound sites, manage exudates, and protect the wound from pathogenic infections by delivering antimicrobial agents. Despite their extensive use, these strategies are unreliable in treating large injuries and chronic wounds, as they rely on slow and passive healing processes (*4*, *5*). To further promote wound healing, much work has focused on the delivery of biologically active agents such as growth factors and cells from the dressing materials (*6*–*9*). Sophisticated wound dressings have been recently developed to monitor and respond to physiological signs from the wound sites, including local strains, temperature, and pH (*10*, *11*). However, these designs are often associated with complex fabrication processes, high cost, drug side effects, and difficulty with the loading and controlled release of the bioactive agents.

**Fig. 1:**
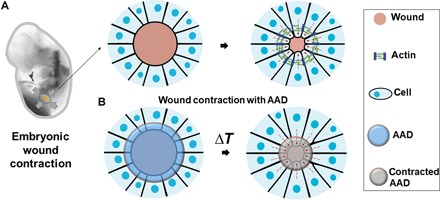
Bioinspired design of AAD for promoting wound contraction. (**A**) Skin wounds of chicken embryo. An actin cable (green) is formed in the cells at the wound edges and contacts the wound. (**B**) Active wound contraction enabled by AAD that adheres to and contracts the wound edges at the skin temperature. Red dashed arrows indicate the contraction.

While existing strategies focus on biochemical functions of wound dressings, much less effort has been exerted to engineer mechanical cues created by the dressings that can promote wound healing. Embryonic wound healing, which provides perfect regeneration of fetal skin, can inspire new strategies for wound dressing design. Embryonic wound healing involves the formation of actin cables at the leading edge of the cells encompassing the wound that contract and apply force to draw the wound edges together in a purse-string–like manner (Fig. 1A) (*12*, *13*). Similar mechanisms aid wound healing in loose-skinned animals such as rodents (*14*). In contrast, the postnatal skin of the adult human exhibits substantially less contraction. Inspired by the contraction ability of embryonic wounds, we propose a new design of wound dressings, called active adhesive dressings (AADs), to exert contractile forces sufficient to promote active wound closure. In this design, AADs are biomechanically active, requiring them to be (i) mechanically robust and tough, (ii) capable of thermoresponsive shrinkage to generate contractile forces after placement on the skin, and (iii) strongly adhesive to efficiently transfer these contractile forces to the underlying wound edges (Fig. 1B). This concept was also inspired by clinical use of negative-pressure wound therapies and the recent demonstration of mechanically driven muscle regeneration (*15*, *16*).

**Fig. 2:**
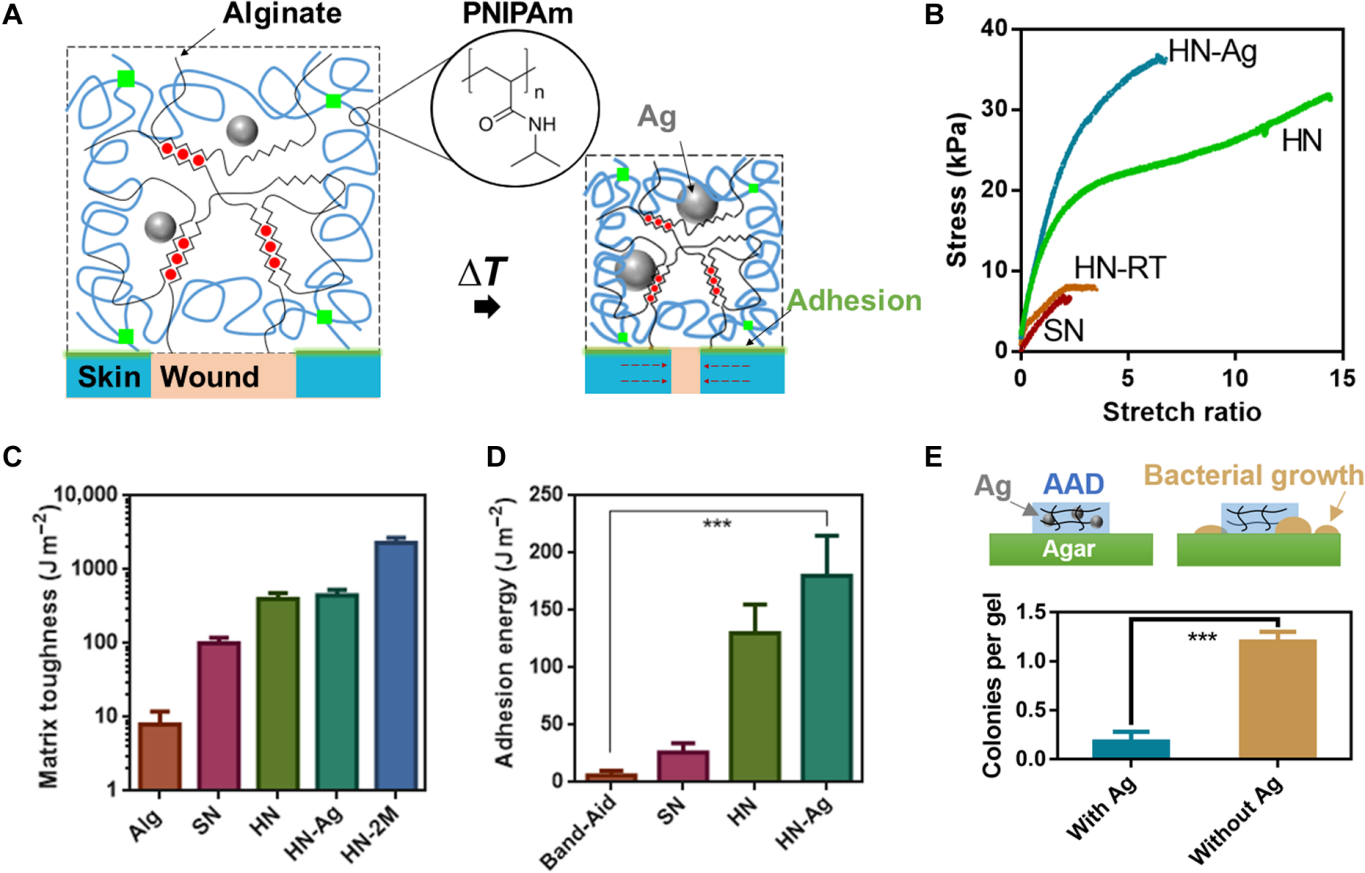
Mechanical and antibacterial properties of AAD. (**A**) Schematics of temperature-triggered transition of AAD, which consist of PNIPAm (blue lines), alginate (black lines), and AgNPs (Ag; grey spheres). AAD forms strong adhesion (green lines) with the wounded skin. (**B**) Stress-strain curves of various hydrogel matrices, including the single-network PNIPAm hydrogel (SN), the hybrid-network PNIPAm-alginate hydrogel (HN), and AgNP-laden hybrid hydrogel (HN-Ag). HN were gelled at 4°C and compared to the same hydrogel form at room temperature (HN-RT). (**C**) Matrix toughness of various hydrogel matrices. Alg refers to alginate hydrogels and 2M refers to 2 M NIPAm monomers used to form HN. Mean ± SD, *n* = 3 to 5. (**D**) Adhesion energy measured on porcine skin. Band-Aid was included for comparison. Mean ± SD, *n* = 3 to 4. (**E**) Antimicrobial function of AAD with and without AgNPs (Ag) (bottom); a schematic of AAD and bacterial growth on agar gels (top). Mean ± SD, *n* = 7, ***P < 0.001.

Whereas current wound dressings cannot satisfy the requirements of AAD, recent progress in tough adhesives (TAs) may enable AAD. TAs have achieved adhesion energies up to 1000 J m^−2^ on various tissues, including skin, even with exposure to blood and dynamic tissue movements (*17*). To restore the wound contraction function of adult skin and avoid wound infection, we proposed to extend the design of TA to create AAD with temperature-triggered contraction and antibacterial function (Fig. 2A). Thermoresponsive behavior was created by fabricating with poly(*N*-isopropyl acrylamide) (PNIPAm); PNIPAm is a widely used thermoresponsive polymer, which repels water and shrinks at around 32°C (*18*, *19*). To transmit the stress to the skin, strong tissue adhesion was achieved via bonding of the adhesive hydrogel to the underlying tissue via chitosan and carbodiimide-mediated reactions, the same method used in TA (*17*). To provide antimicrobial function, silver nanoparticles (AgNPs) were incorporated in the hydrogel matrix. AgNPs have been widely used in wound care products, including commercially available alginate hydrogels (e.g., ALGICELL) and antimicrobial gauze (*20*, *21*). To the best of our knowledge, this is the first design of mechanically active wound dressings for wound management. This new mechanotherapeutic, biological-free approach to accelerating postnatal wound healing may be widely useful in a variety of medical settings.

## RESULTS

A hybrid PNIPAm-alginate network was first engineered for high stretchability and toughness by optimizing the composition and gelation conditions. We discovered that a low gelation temperature and incorporation of an alginate network led to more physically homogeneous, highly stretchable, and tough PNIPAm-based hydrogels. Tensile tests were first performed to determine the stretchability of hydrogels of varied compositions. NIPAm monomer (1 M) was used for the hydrogels unless stated otherwise. These studies demonstrated that the single-network PNIPAm hydrogel (SN) ruptured easily at small strains of <300%. Hybrid-network PNIPAm-alginate hydrogels formed at room temperature (HN-RT) were as brittle as the SN hydrogels (Fig. 2B). In contrast, hybrid hydrogels formed at 4°C (HN) sustained strains of >1400% without rupture, which were much larger than that of the SN counterpart and HN-RT. The HN loaded with AgNPs (HN-Ag) remained highly stretchable and sustained strains of >600% (Fig. 2B).

The high stretchability implied high matrix toughness. Pure-shear tests were performed to quantify the toughness of various hydrogel matrices via calculation of fracture energy, following a previously reported protocol (*17*, *22*–*24*). The hybrid network formed at 4°C (HN) exhibited high toughness (fracture energy, ~500 J m^−2^), whereas the single-network alginate (Alg) or PNIPAm hydrogels (SN) exhibited a much lower fracture energy of <100 J m^−2^ (Fig. 2C). The inclusion of AgNPs had no significant impact on the matrix toughness. With increasing concentration of NIPAm monomers, the fracture energy was further increased beyond 2000 J m^−2^. The results confirmed the synergistic effect of the PNIPAm and alginate networks for superior mechanical performance.

We next explored the adhesion to porcine skin. To form covalent bonds between tissues and the functional groups in the hydrogel matrix, the hydrogel surface was primed with chitosan and carbodiimide coupling agents, following the design of TA (*17*). The chitosan penetrates the skin and the hydrogel, while EDC [1-ethyl-3-(3-dimethylaminopropyl)carbodiimide] and NHS (*N*-hydroxysuccinimide) facilitate the formation of amide bonds between proteins of tissues, chitosan, and alginate within the matrix (*17*). The adhesion energy was quantified with standard 180° peeling adhesion tests. The HN achieved a higher adhesion energy (125 J m^−2^) than the single-network PNIPAm adhesive (SN) (Fig. 2D). The hydrogels with embedded AgNPs HN-Ag achieved slightly higher adhesion energies of around 175 J m^−2^. The adhesion energy was substantially higher than that of clinically used dressings and bandages such as Band-Aid (around 10 J m^−2^).

To test the antimicrobial function of HN, we analyzed bacterial growth on agar plates in the presence of the adhesive hydrogels with and without AgNPs (fig. S1). AgNP-containing HN-Ag effectively inhibited bacterial growth (Fig. 2E). To examine whether AgNPs were lost from HN-Ag over time, we performed a release study. We found no AgNPs that leaked from the hydrogels, indicating that the antimicrobial function of HN-Ag relied on the release of silver ions, not of nanoparticles, from the gels.

**Fig. 3:**
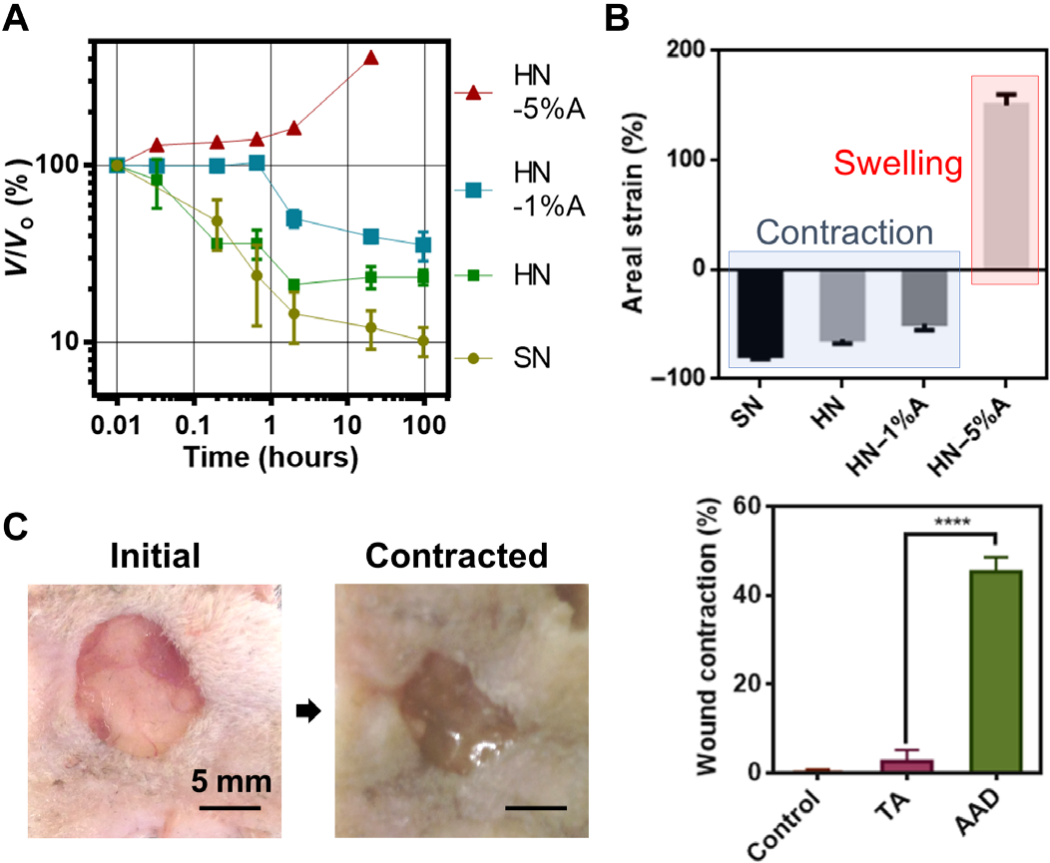
Thermoresponsiveness of AAD and in vitro contraction on skin. (**A**) Thermoresponsive behavior varies with the matrix composition of HN. 1%A and 5%A refer to 1% and 5% of acrylamide within the total monomer. SN represents PNIPAm-only gel used as a control. (**B**) Areal strains as a function of the matrix composition measured under equilibrium conditions at 37°C. Negative values of the areal strain indicate contraction, whereas positive values indicate expansion. Mean ± SD, *n* = 3 to 4. (**C**) In vitro tests of AAD-enabled wound contraction on fresh rodent skin. Comparison of the wound area between nontreatment control, nonthermoresponsive dressing TA, and AAD. Mean ± SD, *n* = 5, ****P < 0.0005. Photo credit: Jianyu Li and David Mooney, Harvard University.

We next characterized the temperature-triggered contraction behavior of the adhesive hydrogels and explored a strategy to modulate the thermoresponsive behavior via copolymerizing acrylamide monomers (AAm). We hypothesized that the inclusion of AAm could tune the contractile behavior of the PNIPAm-based hydrogels. The PNIPAm-alginate hydrogels contracted to ~20% of their initial volume within 3 hours when placed at 37°C, resulting in an areal contractile strain of 64% (Fig. 3A). This was comparable with that observed for the single-network PNIPAm gels. The areal contractile strain varied with the relative contents of AAm and NIPAm; a small fraction of AAm (1%) reduced the contractile strain to 46%, whereas an AAm content of 5% led to swelling of the resulting matrices (Fig. 3B). The ability to tune the active response is expected to allow one to program the strain imposed on wound edges.

We next explored the use of these thermoresponsive adhesive hydrogels, termed AAD. In vitro experiments demonstrated that AAD can apply contractile strains to wound edges on the skin following temperature-triggered contraction. We placed AAD on explanted rodent skin in which a circular defect was created to mimic a full-thickness skin wound (Fig. 3C). The specimens were placed at 37°C overnight and then flash-frozen with liquid nitrogen to measure the wound size. This study included nontreatment controls and TA consisting of a nonthermoresponsive polyacrylamide-alginate matrix. The results showed that AAD reduced the wound area to around 45%, compared to minimal changes in the nontreatment control and the TA condition (Fig. 3C).

**Fig. 4:**
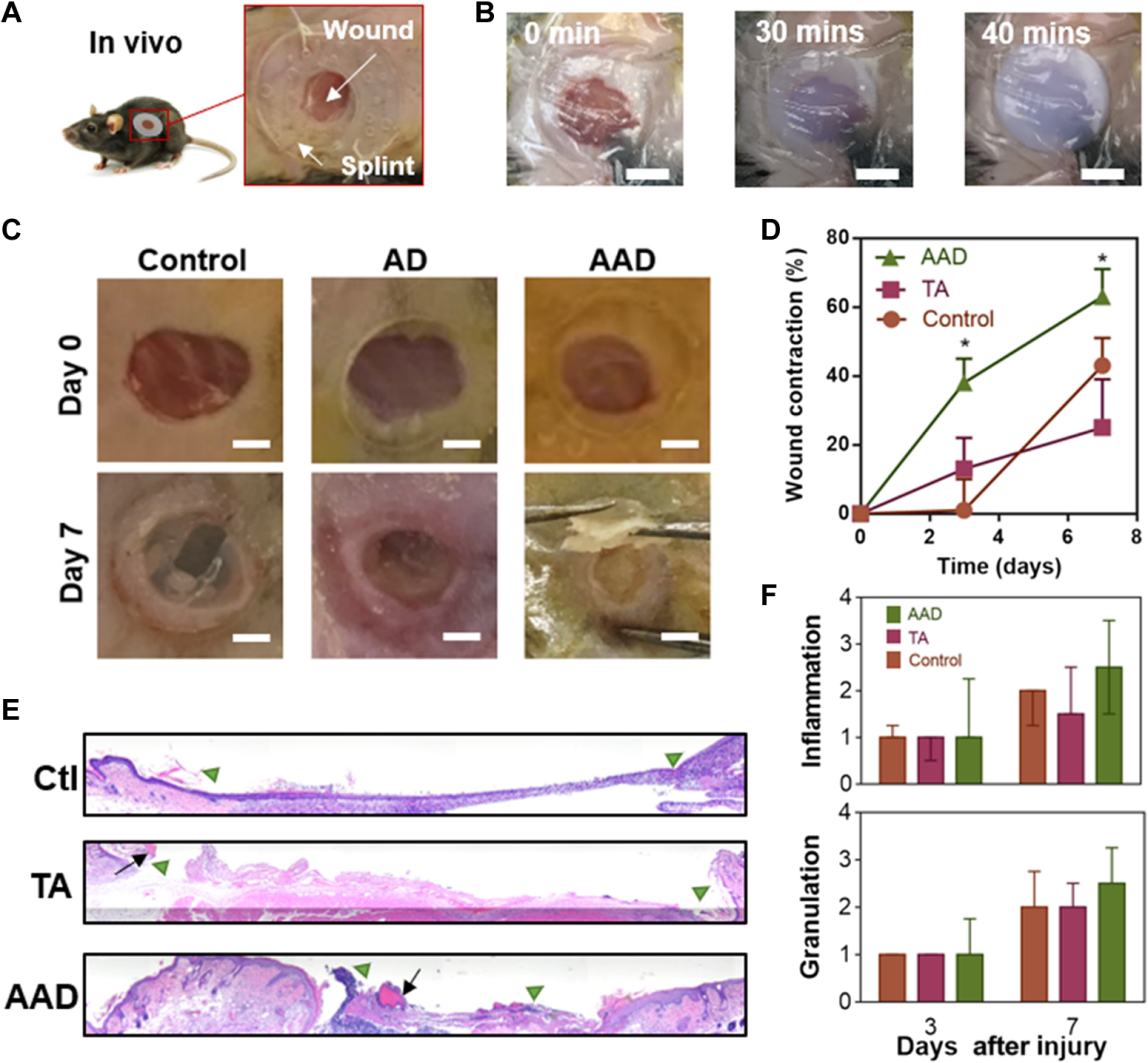
In vivo wound healing with application of AAD. (**A**) In vivo rodent wounds with splints. (**B**) Skin temperature–triggered response of AAD on a skin wound. Scale bars, 5 mm. (**C**) Digital images of the initial wounds and those after 7 days with no hydrogel treatment (control), with treatment with nonthermoresponsive TA, and with treatment with AAD. Scale bars, 2 mm. (**D**) Wound contraction as function of time and treatments. Mean ± SD, *n* = 5, *P < 0.05. (**E**) Histological sections of the wounded skin harvested on day 7 and hematoxylin and eosin–stained. The wound edges and the residues of adhesives are marked with green triangles and black arrows, respectively. (**F**) Histological assessment of levels of inflammation and granulation by a blinded pathologist expert (0, normal; 1, minimal; 2, mild; 3, moderate; and 4, strong). Median ± interquartile range, *n* = 3 to 5 per group. Photo credit: Jianyu Li and David Mooney, Harvard University.

To validate the efficacy of AAD in vivo, we placed AAD over rodent skin wounds. We used a murine skin wound healing model in which a rubber splint is fixed to the wound edges (Fig. 4A); this model limits the normal wound contraction of rodents to better simulate the native healing response of humans and fixed-skinned mammals (*14*, *25*). We further covered the wound sites with Tegaderm (3M Inc.) and Band-Aid (Johnson & Johnson Inc.) to minimize water loss and protect from animal scratching, respectively. This test included TA and nontreatment control conditions for comparison. In the nontreatment control condition, the wound sites were directly covered with Tegaderm dressing. Upon contact of AAD with skin, a phase transition of the AAD occurred within 40 min as manifested by a transparency change (Fig. 4B). Wound size measurements showed that the AAD significantly accelerated wound closure, compared to nontreatment control and TA as early as day 3 (Fig. 4, C and D). A significant improvement of wound closure with AAD was found on day 7, as compared to the other conditions; no significant difference in wound closure were found between TA and nontreatment control. To assess the levels of inflammation and granulation tissues with the various treatments, the dressings and the surrounding tissues were harvested on days 3 and 7, fixed, and stained for histological assessment (Fig. 4E and fig. S2). AAD facilitated granulation tissue formation and re-epithelialization of the wounds, and all conditions exhibited similar levels of inflammation and no severe immune response, indicative of good biocompatibility (Fig. 4F).

**Fig. 5:**
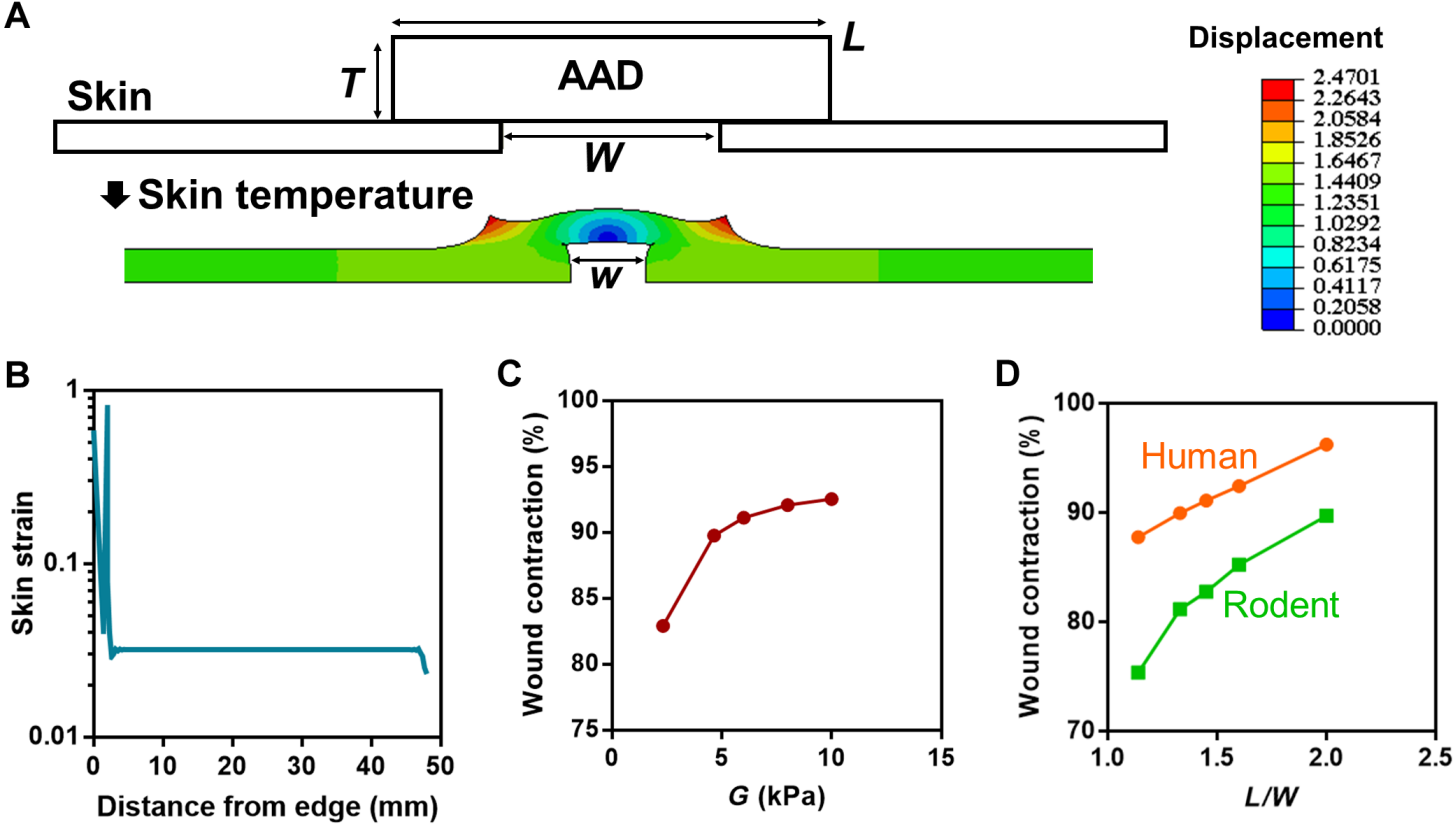
Finite element simulation of AAD-enabled wound contraction. (**A**) Configuration of the finite element model where AAD of thickness *T*, width *L*, and shear modulus *G* is attached onto a wounded skin of initial wound size *W* (top). After contraction, the wound size is reduced to *w* (bottom). The color contours map the magnitude of horizontal displacement. (**B**) Simulated rodent skin strain as a function of the distance from the wound edge. (**C**) The wound contraction, calculated by 1 − (*w*/*W*)^2^, is predicted to vary with the shear modulus *G* of AAD. (**D**) Modeling of contraction of human and rodent skin as a function of the length of AAD to width of the wound ratio (*L*/*W*).

Last, to further characterize the mechanical interaction between the wounded skin and AAD and to develop an in silico platform to optimize the closure process, we developed finite element models to simulate the interaction between AAD and the wounded skin using the commercial software ABAQUS (Fig. 5A). AAD was simulated with a modified Flory-Rehner model (*19*), which models the highly nonlinear thermoresponse of PNIPAm hydrogels. The skin was modeled as an Ogden hyperelastic material, which successfully captured the initial toe modulus and stiffening effect of skin under large strains in our measurements (fig. S3). The finite element models were parameterized with our mechanical characterization of the rodent skin and AAD and the values extracted from the literature for simulating thermoresponse of PNIPAm (table S1) (*19*). The simulation showed a clear wound closure accompanying the contraction of AAD, as evidenced by finite displacements and strains developed in the wounded skin substrate (Fig. 5B, fig. S3, and movie S1). Given the properties of the AAD and rodent skin, the simulated wound contraction (around 75%) was comparable with the experimentally measured value (63 ± 8%). This model was then used to investigate how the material and geometric properties of AAD affect wound contraction. Our simulations predicted that wound contraction increased with increasing modulus of AAD (Fig. 5C) and with increasing adhesion area between AAD and skin (Fig. 5D). In addition, a simulation performed using the mechanical properties of human skin predicted that AAD could induce human skin contraction comparable to that of rodent skin (*26*).

## DISCUSSION

In this work, we designed and fabricated an AAD based on a tough, adhesive, and thermoresponsive hydrogel. The AAD combined superior stretchability and toughness, attributing to low-temperature gelation and the double-network design. Lowering the gelation temperature improved the mixing of the hydrogel precursors, resulting in more homogenous hydrogels. Phase separation of the PNIPAm from alginate occurred at room temperature, presumably due to the lower hydrophilicity of NIPAm compared to acrylamide (*18*, *19*). The double-network design created a synergy between the two cross-linked networks: The covalently cross-linked PNIPAm network bridges the crack tip and preserves the overall integrity of the matrix, while the ionically cross-linked alginate network dissipates energy under deformation. Given the same concentration of covalent cross-linkers, raising the monomer content is expected to increase the chain length of PNIPAm between two covalent cross-links, leading to a more prominent crack-bridging effect and thus larger fracture energy. These results were consistent with previous findings on tough polyacrylamide-alginate hydrogels and other double-network hydrogels (*22*–*24*).

The AAD demonstrated temperature-triggered contraction and strong adhesion to the skin. Few efforts have been made to fabricate thermoresponsive adhesives. In previous studies, researchers used thermoresponsive polymers predominately for network gelation or to control swelling (*27*, *28*). For instance, the sol-gel transition of Pluronic F127 (Plu) was used to drive gelation of dopamine-modified hyaluronic acid/Plu adhesive hydrogels when exposed to the body temperature (*27*), and thermoresponsive poly(propylene oxide)-poly(ethylene oxide) copolymers were incorporated into mussel-inspired surgical adhesives to control the swelling of the adhesives in the body (*28*).

**Fig. 6:**
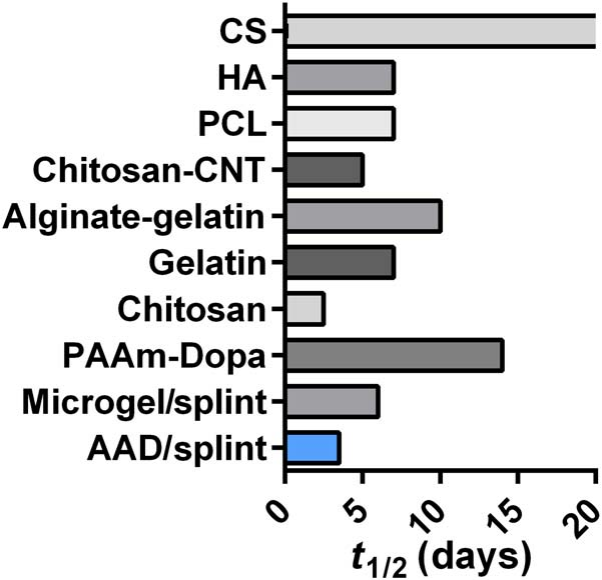
Wound closure rate, as characterized by the time for 50% wound closure (*t*_1/2_) with various reported wound dressings. Comparisons include AAD (data reported in Fig. 4), microgels (*25*), chitosan (*35*), dopamine-modified polyacrylamide (PAAm-Dopa) (*50*), gelatin (*51*), alginate-gelatin hydrogels (*52*), carbon nanotube–laden chitosan hydrogels (chitosan-CNT) (*53*), polycaprolactone nanofibrous matrix (PCL) (*54*), hyaluronan (HA), and chondroitin sulfate (CS) hydrogels (*55*). The half-life time (*t*_1/2_) resulting from chondroitin sulfate hydrogels was beyond 20 days. While AAD and microgels were tested in a rodent wound model with splints, most previous reports did not use splinting.

This work may open new avenues for developing wound dressings based on adhesive and stimuli-responsive hydrogels. The in vitro and in vivo studies demonstrated that the AAD actively contracted wounds and accelerated wound healing. The active wound contraction of AAD takes advantages of the intrinsic temperature change during placement of a dressing onto the body and requires no additional reagent or sophisticated apparatus for external stimuli [e.g., acid, vacuum, and ultraviolet (UV) light]. Recent years have witnessed numerous surgical adhesives being used in clinics (*29*) and active development of bioinspired adhesives, such as mussel-inspired adhesives (*27*, *28*), gecko-inspired adhesives (*30*), and topologically designed adhesives (*31*). There exist a variety of strategies and hydrogel systems responsive to various stimuli, including light, ionic strength, glucose, and pH (*32*, *33*). In addition, various strategies have been developed to engineer hydrogels for cell and drug delivery, and these can enable biologically active wound healing (*9*, *34*). To compare with other wound dressings under development, we tabulated the wound half-life (i.e., the time required to achieve 50% wound areal contraction) of various wound dressings (Fig. 6). This plot was based on experimental values extracted from reports in which the dressings contained no biological agents. The rate of wound closure with AAD was comparable to that of photo–cross-linked chitosan hydrogels and microporous gel scaffolds reported recently (*25*, *35*). Notably, many studies have not used a splinted wound model, and the rapid wound contraction of rodent skin may complicate interpretation of studies not using splinting. We also compare AAD, TA (*17*), and the key components of commercially available products related to wound care, including skin graft (*36*), ALGICELL (Derma Sciences) (*17*), Band-Aid (Johnson & Johnson), Tegaderm (3M) (*37*, *38*), COSEAL (*17*), TISSEEL (Baxter) (*39*), and DERMABOND (Ethicon) (*17*). Table S2 shows that the AAD exhibits a unique combination of adhesiveness, toughness, and antimicrobial and active contraction properties, which enables the wound contraction and accelerates the wound healing process.

Previous studies have introduced the concept of mechanoactive materials for tissue regeneration. These approaches achieve material actuation by incorporating a degradable network within aligned poly(lactic-co-glycolic acid) nanofibers (*40*), breaking catalyst-bearing microstructures within hydrogels (*41*), applying a magnetic field to hydrogels containing iron oxide nanoparticles (*42*), using ultrasound to disrupt ionic bonds in ionically cross-linked hydrogels (*43*), and using optics to optothermally actuate polyethylene glycol diacrylate hydrogels (*44*). Often, these strategies require external stimuli not present in the host environment to promote material actuation. Therefore, our AAD may be advantageous since it relies on native tissue temperature for mechanoactivation.

Although our work has identified unprecedented mechanical performance of the AAD and its ability to promote wound closure in mice, much work is still required to evaluate the effect of the AAD on skin regeneration. For example, future studies will evaluate the impact on the expression of genes known to be important in wound healing and collagen organization in healing skin tissue over time. It will be important to elucidate how the mechanical cues exerted by AAD affect the biological process of wound healing [e.g., the phenotype, migration, and activity of relevant cells such as fibroblasts (*45*)].

Further investigation is needed to explore the ability of finite element modeling to optimize AAD and to examine the function of AAD under a wide range of environmental temperature. The finite element model can be further improved by accounting for in vivo boundary conditions [two-dimensional (2D) versus 3D] and biological processes (*46*, *47*) to provide greater predictive values under various clinical scenarios. To date, we have only studied the contraction of the materials at normal body temperatures (35° to 37°C). Future studies will examine the temperature dependence of contraction in our AAD, as cold ambient temperatures may require the skin to be warmed when the AAD is applied. A previous study has demonstrated that the temperature of the skin can vary at different locations on the body (*48*, *49*), and future studies are necessary to identify whether regions of cooler body temperature enable AAD contraction.

In conclusion, we developed a novel design of mechanotherapeutic dressings that may create a new paradigm for wound management. Inspired by embryonic wound contraction and exploiting recent advances in hydrogels and adhesives, this work led to AAD with an unprecedented combination of mechanical, biological, and antibacterial properties. The AAD forms strong adhesion to skin and generates sufficient contractile strains, in response to exposure to skin temperature to enhance healing. A finite element model suggests avenues to further program the performance of AAD and the feasibility of using AAD on human skin. These new materials may find broad utility in the field of regenerative medicine, as they may be similarly useful in treatment of wounds in other epithelial tissues such as intestine, lung, and liver. They may also be useful in drug delivery and as components of soft robotics-based therapies.

## MATERIALS AND METHODS

### Synthesis and implementation of AAD

Chemical reagents used in this study were purchased from Sigma-Aldrich unless stated otherwise. The alginate was PRONOVA UP MVG purchased from FMC Corporation. Both nonthermoresponsive and thermoresponsive TA and AAD hydrogels were tested as wound dressings in this work. The nonthermoresponsive adhesives, called TA, consisted of alginate-polyacrylamide hydrogels, chitosan, and coupling reagents (e.g., EDC and NHS) (*17*). The thermoresponsive adhesives, called AAD, consist of a bilayered structure: a thermoresponsive antimicrobial dissipative matrix and a tissue-adhesive surface. The matrix was a PNIPAm-alginate hybrid network with embedded Ag-NPs for antimicrobial function, which was formed using *N*,*N*′-methylenebisacrylamide (MBAA) to covalently cross-link NIPAm and calcium sulfate (CaSO_4_) to ionically cross-link alginate. Briefly, 1.06 M NIPAm/0.083 M alginate solution in phosphate-buffered saline (PBS) was syringe mixed with 0.28 mM MBAA, 0.02 M CaSO_4_, 0.0065 M initiator ammonium persulfate, and 0.0037 M accelerator tetramethylethylenediamine. For antimicrobial function, 4 mg of AgNPs (diameter, 30 to 50 nm; US Research Nanomaterials Inc.) was mixed into the hydrogel precursors before gelation. For the hydrogels containing acrylamide (AAm), the molar percentages of AAm within the entire monomers (AAm plus NIPAm) were varied (0, 1, and 5%), as indicated above. The hydrogels were gelled inside a closed glass mold at either 4° or 20°C overnight. The adhesive surface was achieved by activating the hydrogel surfaces with chitosan (medium molecular weight) and carbodiimide reagents (e.g., EDC and NHS) according to a previously reported protocol (*17*). The concentration of chitosan was 2% (w/v); EDC and NHS were both 12 mg/ml. The mixture of chitosan, EDC, and NHS was applied to the surface of hydrogel matrix before application. For the hydrogels used in the in vivo experiments, the alginate and chitosan were sterile-filtered, frozen, and lyophilized for 1 week. All the other chemical agents such as NIPAm, AAm, and EDC were sterilized by filtering right before usage. The synthesis was conducted in a tissue culture hood. After gelation, the hydrogels were soaked in saline for 30 min and then rinsed three times before usage. The materials were stored at 4°C before use.

### Pure-shear test

Pure-shear tests were performed following protocols reported previously (*17*, *24*). An Instron machine (model 3342 with a load cell maximum of 50 N) was used to apply unidirectional tension on specimens (80 mm by 5 mm by 1.5 mm). The strain limit was defined as the ratio of the extension of the specimen upon rupture to that of the undeformed specimen. The elastic modulus was defined as the tangent of the stress-stretch curve at small strains (<10%). The fracture energy was determined by the stress-stretch curves of unnotched and notched specimens following a previously reported protocol (*17*, *24*).

### Peeling adhesion test

The same Instron machine was used to perform standard 180° peeling adhesion tests to quantify the adhesion performance (*17*). The adhesion energy was calculated by 2 × *F*/*w*, where *F* is the plateau force during peeling and *w* is the width of the specimen. The tissues used in the peeling tests included porcine skin and fresh explanted rodent skin (C57BL/6J mice; female, aged 6 to 8 weeks; the Jackson Laboratory). The peeling rate was fixed at 100 mm/min.

### Thermoresponse characterization

The thermoresponse was evaluated by placing the specimens in PBS at 37°C and measuring the volume change over time. The initial and final dimensions were denoted as *L*_o_ and *L*, and the areal strain was calculated by 1 − (*L*_o_/*L*)^2^.

### Antimicrobial characterization

Skin bacteria were added to the surface of agar plates with one of the following treatments applied: nontreatment control, pristine sliver nanoparticles (AgNPs), and AAD with or without AgNPs. Plates were placed at 37°C, and bacterial growth was visualized in 3 days. For the encapsulation study, AgNP-laden AAD (diameter, 10 mm; thickness, 1.5 mm) was placed in 1 ml of Dulbecco’s phosphate-buffered saline (DPBS) solution at 37°C. The buffer was collected and replaced at certain time points (0.5, 2, 8, 24, and 48 hours and 7 days). A UV/visual spectroscopy with an absorbance at 420 nm was used to quantify the amount of AgNP released from AAD, and the calibration curve was measured with AgNP standards of 1, 0.5, 0.25, 0.125, 0.0625, and 0.03125 mg/ml. At every time point, there was no AgNP found in the release medium within the detectable range.

### In vitro wound closure experiments

Wound closure was tested in vitro on fresh explanted rodent skin (female C57BL/6J mice, aged 6 to 8 weeks; the Jackson Laboratory). AAD (15 mm by 15 mm by 1.5 mm) was applied to skin with 10-mm-diameter wounds and gently compressed at room temperature for 15 min. The skin and AAD were then placed at 37°C for 2 days and then frozen in liquid nitrogen. The AAD was then removed, and the wound was photographed to determine the wound size.

### Finite element simulation

Finite element simulation was conducted with a commercially available software ABAQUS (ABAQUS Inc., version 2017). A user-defined subroutine was used to simulate the thermoresponsive behavior of AAD following a previously reported protocol (*19*). Briefly, a modified Flory-Rehner free-energy function was used for implementing nonlinear thermoresponse of AAD by using a user-defined UHYPER subroutine in ABAQUS. The skin was modeled as an Ogden hyperelastic material via the equation

$$s=\mu (\lambda^{\alpha }-\lambda^{-\alpha /2})$$

where α and μ are two Ogden’s coefficients, *s* is nominal stress (i.e., the measured force divided by the cross-sectional area of an undeformed specimen), and λ is the stretch ratio. For human skin, α and μ are 10 and 110 Pa, respectively, cited from (*26*). The two material constants were obtained from the fitting of stress-strain curves of fresh dorsal skin of C57BL/6J mice (female, aged 6 to 8 weeks; the Jackson Laboratory) measured with tensile tests (see fig. S3B and table S1); the fitting was conducted at stretch ratios of <1.6, beyond which the strain-strain curves showed zigzag shapes indicative of rupture or grip debonding. The shear modulus of AAD was determined with *E*/2(1 + *v*), where *E* is the elastic modulus measured from tensile tests and *v* is the Poisson’s ratio 0.5 given the incompressibility of the hydrogel. The other parameters required to formulate the thermoresponse were based on previous studies on PNIPAm hydrogels (table S1) (*19*), as PNIPAm was responsible for the thermoresponse of AAD. In the simulation, the AAD and skin were modeled as a 2D system because of symmetry, using eight-node biquadratic plane strain quadrilateral hybrid elements; the width of the skin was 100 mm estimated from the circumference of the mice used in the in vivo study. The width of AAD and wound were 8 and 4 mm, respectively, unless stated otherwise. The far left and right edge of the skin was fully fixed, while the horizontal displacement of the bottom was fixed (fig. S3). The AAD-skin system was warmed up from the initial temperature of 280 to 310 K to trigger the active response of AAD. The measured thickness of skin was 0.5 mm, while the thickness, width, and shear modulus of AAD was varied, as indicated in Fig. 5 and fig. S3. The wound contraction was calculated by 1 − (*w*/*W*)^2^, where *w* and *W* are the wound sizes after and before contraction, respectively (Fig. 5A).

### In vivo studies

Full-thickness excision wound healing experiments were conducted based on a reported protocol for limiting spontaneous rodent wound contraction (*14*, *25*). C57BL/6J mice (female, aged 6 to 8 weeks; the Jackson Laboratory) were used for in vivo studies. All the experiments were carried out in accordance with the Institutional Animal Care and Use Committee at Harvard University and institutional guidelines of the National Institute of Health. Anesthesia of mice was induced with isoflurane (3 to 4%) and then maintained with continuous flow of isoflurane (1 to 2%). Hair on the dorsal surface of the skin was shaved, removed with depilatory cream, and prepared aseptically. A full-thickness dorsal excisional skin wound was created on mice with a sterile 8-mm-diameter biopsy punch following removal of hair. A rubber splint to prevent native wound closure was made with polydimethylsiloxane with inner and outer diameters of 12 and 20 mm, respectively. The splint was centered around the wound and fixed with super glue and four interrupted sutures to secure the position. An AAD or TA with a diameter of 10 mm was then placed on the wound inside the rubber splint and compressed for 2 min with a custom-made applicator containing ice. Nontreatment controls were included for comparison. A Tegaderm (3M Inc) and a bandage were then applied to avoid dehydration due to water evaporation and to prevent scratching or biting on the specimen, respectively. After the mice recovered from anesthesia, they were placed back into the cage and monitored daily to assess health. The dressings and the surrounding tissue were harvested on days 3 and 7 for histological assessments. Each condition has five mice at each time point.

### Histology assessment

The explanted samples were fixed with 4% paraformaldehyde/PBS at 4°C overnight, followed by PBS rinsing three times, and then processed for hematoxylin and eosin staining at the Rodent Pathology Core at Harvard Medical School. The histological sections were imaged with a Nikon E800 upright microscope. A blinded histopathology expert evaluated the degree of inflammation and granulation tissue formation, which was scored with a four-point scale (0, normal; 1, minimal; 2, mild; 3, moderate; and 4, strong).

### Statistics

Statistical analysis in this study was performed using embedded algorithms in the commercial software GraphPad Prism 6. One-way analysis of variance (ANOVA) was used to analyze multiple sets of data, while Student’s *t* tests was used to analyze experiments involving two sets of data. Two-way nonparametric ANOVAs were used for the healing study (time and dressing) and was performed in SPSS Statistics (IBM). The sample sizes *n*, means, and SDs are shown in the figure legends. The *P* values were calculated by ANOVA or Student’s *t* test. The levels of significance are labeled with *P ≤ 0.05, **P ≤ 0.01, ***P ≤ 0.005, and ****P ≤ 0.0005.

## Supplementary Material

http://advances.sciencemag.org/cgi/content/full/5/7/eaaw3963/DC1

Download PDF

Movie S1

## SUPPLEMENTARY MATERIALS

Supplementary material for this article is available at http://advances.sciencemag.org/cgi/content/full/5/7/eaaw3963/DC1 Fig. S1. Antimicrobial tests. Fig. S2. Histological sections on day 3 specimens. Fig. S3. Finite element simulation. Table S1. Material parameters used in finite element stimulation. Table S2. Comparison of mechanical and antimicrobial properties of materials related to wound care. Movie S1. Finite element simulation of AAD-enabled wound contraction.

## References

[1] Singer A. J., Clark R. A. F., Cutaneous wound healing. N. Engl. J. Med. 341, 738–746 (1999). 10471461 10.1056/NEJM199909023411006

[2] Fong E., Tirrell D. A., Collective cell migration on artificial extracellular matrix proteins containing full-length fibronectin domains. Adv. Mater. 22, 5271–5275 (2010). 20886461 10.1002/adma.201002448 PMC3027490

[3] Zhu S., Nih L., Carmichael S. T., Lu Y., Segura T., Enzyme-responsive delivery of multiple proteins with spatiotemporal control. Adv. Mater. 27, 3620–3625 (2015). 25962336 10.1002/adma.201500417 PMC4633528

[4] Dyson M., Young S., Pendle C. L., Webster D. F., Lang S. M., Comparison of the effects of moist and dry conditions on dermal repair. J. Invest. Dermatol. 91, 434–439 (1988). 3171219 10.1111/1523-1747.ep12476467

[5] Yannas I. V., Burke J. F., Orgill D. P., Skrabut E. M., Wound tissue can utilize a polymeric template to synthesize a functional extension of skin. Science 215, 174–176 (1982). 7031899 10.1126/science.7031899

[6] Boateng J. S., Matthews K. H., Stevens H. N. E., Eccleston G. M., Wound healing dressings and drug delivery systems: A review. J. Pharm. Sci. 97, 2892–2923 (2008). 17963217 10.1002/jps.21210

[7] Johnson N. R., Wang Y., Drug delivery systems for wound healing. Curr. Pharm. Biotechnol. 16, 621–629 (2001). 10.2174/1389201016666150206113720 PMC6053062 25658378

[8] Wu Y., Chen L., Scott P. G., Tredget E. E., Mesenchymal stem cells enhance wound healing through differentiation and angiogenesis. Stem Cells 25, 2648–2659 (2007). 17615264 10.1634/stemcells.2007-0226

[9] Li J., Mooney D. J., Designing hydrogels for controlled drug delivery. Nat. Rev. Mater. 1, 16071 (2016). 29657852 10.1038/natrevmats.2016.71 PMC5898614

[10] Lin S., Yuk H., Zhang T., Parada G. A., Koo H., Yu C., Zhao X., Stretchable hydrogel electronics and devices. Adv. Mater. 28, 4497–4505 (2016). 26639322 10.1002/adma.201504152 PMC4896855

[11] Mirani B., Pagan E., Currie B., Siddiqui M. A., Hosseinzadeh R., Mostafalu P., Zhang Y. S., Ghahary A., Akbari M., An advanced multifunctional hydrogel-based dressing for wound monitoring and drug delivery. Adv. Healthc. Mater. 6, 1700718 (2017). 10.1002/adhm.201700718 PMC5845857 28944601

[12] Nodder S., Martin P., Wound healing in embryos: A review. Anat. Embryol. (Berl) 195, 215–228 (1997). 9084820 10.1007/s004290050041

[13] Martin P., Lewis J., Actin cables and epidermal movement in embryonic wound healing. Nature 360, 179–183 (1992). 1436096 10.1038/360179a0

[14] Wang X., Ge J., Tredget E. E., Wu Y., The mouse excisional wound splinting model, including applications for stem cell transplantation. Nat. Protoc. 8, 302–309 (2013). 23329003 10.1038/nprot.2013.002

[15] Thompson J. T., Marks M. W., Negative pressure wound therapy. Clin. Plast. Surg. 34, 673–684 (2007). 17967622 10.1016/j.cps.2007.07.005

[16] Cezar C. A., Roche E. T., Vandenburgh H. H., Duda G. N., Walsh C. J., Mooney D. J., Biologic-free mechanically induced muscle regeneration. Proc. Natl. Acad. Sci. U.S.A. 113, 1534–1539 (2016). 26811474 10.1073/pnas.1517517113 PMC4760832

[17] Li J., Celiz A. D., Yang J., Yang Q., Wamala I., Whyte W., Seo B. R., Vasilyev N. V., Vlassak J. J., Suo Z., Mooney D. J., Tough adhesives for diverse wet surfaces. Science 357, 378–381 (2017). 28751604 10.1126/science.aah6362 PMC5905340

[18] Hirokawa Y., Tanaka T., Volume phase transition in a nonionic gel. J. Chem. Phys. 81, 6379–6380 (1984).

[19] Cai S., Suo Z., Mechanics and chemical thermodynamics of phase transition in temperature-sensitive hydrogels. J. Mech. Phys. Solids 59, 2259–2278 (2011).

[20] Maneerung T., Tokura S., Rujiravanit R., Impregnation of silver nanoparticles into bacterial cellulose for antimicrobial wound dressing. Carbohydr. Polym. 72, 43–51 (2008).

[21] Varaprasad K., Mohan Y. M., Vimala K., Raju K. M., Synthesis and characterization of hydrogel-silver nanoparticles-curcumin composites for wound dressing and antibacterial application. J. Appl. Polym. Sci. 121, 784–796 (2013).

[22] Zhao X., Multi-scale multi-mechanism design of tough hydrogels: Building dissipation into stretchy networks. Soft Matter 10, 672–687 (2014). 24834901 10.1039/C3SM52272E PMC4040255

[23] Li J., Suo Z., Vlassak J. J., Stiff, strong, and tough hydrogels with good chemical stability. J. Mater. Chem. B 2, 6708–6713 (2014). 10.1039/c4tb01194e 32261867

[24] Sun J.-Y., Zhao X., Illeperuma W. R. K., Chaudhuri O., Oh K. H., Mooney D. J., Vlassak J. J., Suo Z., Highly stretchable and tough hydrogels. Nature 489, 133–136 (2012). 22955625 10.1038/nature11409 PMC3642868

[25] Griffin D. R., Weaver W. M., Scumpia P. O., Di Carlo D., Segura T., Accelerated wound healing by injectable microporous gel scaffolds assembled from annealed building blocks. Nat. Mater. 14, 737–744 (2015). 26030305 10.1038/nmat4294 PMC4615579

[26] Mahmud J., Holt C., Evans S., Manan N. F. A., Chizari M., A parametric study and simulations in quantifying human skin hyperelastic parameters. Procedia Eng. 41, 1580–1586 (2012).

[27] Lee Y., Chung H. J., Yeo S., Ahn C.-H., Lee H., Messersmith P. B., Park T. G., Thermo-sensitive, injectable, and tissue adhesive sol–gel transition hyaluronic acid/pluronic composite hydrogels prepared from bio-inspired catechol-thiol reaction. Soft Matter 6, 977–983 (2010).

[28] Darrett D. G., Bushnell G. G., Messersmith P. B., Mechanically robust, negative-swelling, mussel-inspired tissue adhesives. Adv. Healthc. Mater. 2, 745–755 (2013). 23184616 10.1002/adhm.201200316 PMC3685437

[29] Duarte A. P., Coelho J. F., Bordado J. C., Cidade M. T., Gil M. H., Surgical adhesives: Systematic review of the main types and development forecast. Prog. Polym. Sci. 37, 1031–1050 (2012).

[30] Mahdavi A., Ferreira L., Sundback C., Nichol J. W., Chan E. P., Carter D. J. D., Bettinger C. J., Patanavanich S., Chignozha L., Ben-Joseph E., Galakatos A., Pryor H., Pomerantseva I., Masiakos P. T., Faquin W., Zumbuehl A., Hong S., Borenstein J., Vacanti J., Langer R., Karp J. M., A biodegradable and biocompatible gecko-inspired tissue adhesive. Proc. Natl. Acad. Sci. U.S.A. 105, 2307–2312 (2008). 18287082 10.1073/pnas.0712117105 PMC2268132

[31] Yang J., Bai R., Suo Z., Topological adhesion of wet materials. Adv. Mater. 30, 1800671 (2018). 10.1002/adma.201800671 29726051

[32] Qiu Y., Park K., Environment-sensitive hydrogels for drug delivery. Adv. Drug Deliv. Rev. 64, 49–60 (2012). 10.1016/s0169-409x(01)00203-4 11744175

[33] Brudno Y., Mooney D. J., On-demand drug delivery from local depots. J. Control. Release 219, 8–17 (2015). 26374941 10.1016/j.jconrel.2015.09.011

[34] Whittam A. J., Mann Z. N., Duscher D., Wong V. W., Barrera J. A., Januszyk M., Gurtner G. C., Challenges and opportunities in drug delivery for wound healing. Adv. Wound Care 5, 79–88 (2016). 10.1089/wound.2014.0600 PMC4742986 26862465

[35] Ishihara M. I., Nakanishi K., Ono K., Sato M., Kikuchi M., Saito Y., Yura H., Matsui T., Hattori H., Uenoyama M., Kurita A., Photocrosslinkable chitosan as a dressing for wound occlusion and accelerator in healing process. Biomaterials 23, 833–840 (2002). 11771703 10.1016/s0142-9612(01)00189-2

[36] Dong C., Mead E., Skalak R., Fung Y. C., Debes J. C., Zapata-Sirvent R. L., Andree C., Greenleaf G., Cooper M., Hansbrough J. F., Development of a device for measuring adherence of skin grafts to the wound surface. Ann. Biomed. Eng. 21, 51–55 (1993). 8434820 10.1007/BF02368164

[37] Li L., Tirrell M., Korba G. A., Pocius A. V., Surface energy and adhesion studies on acrylic pressure sensitive adhesives. J. Adhes. 76, 307–334 (2001).

[38] Pharr M., Sun J.-Y., Suo Z., Rupture of a highly stretchable acrylic dielectric elastomer. J. Appl. Phys. 111, 104114 (2012).

[39] Kjaergard H. K., Velada J. L., Pulawska T., Ellensen V. S., Larsen S. S., Hollingsbee D. A., Development of a model for measurement of adhesion strength of fibrin sealant to human tissue. Eur. Surg. Res. 31, 491–496 (1999). 10861345 10.1159/000008729

[40] Spalazzi J. P., Vyner M. C., Jacobs M. T., Moffat K. L., Lu H. H., Mechanoactive scaffold induces tendon remodeling and expression of fibrocartilage markers. Clin. Orthop. Relat. Res. 466, 1938–1948 (2008). 18512112 10.1007/s11999-008-0310-8 PMC2584247

[41] He X., Aizenberg M., Kuksenok O., Zarzar L. D., Shastri A., Balazs A. C., Aizenberg J., Synthetic homeostatic materials with chemo-mechano-chemical self-regulation. Nature 487, 214–218 (2012). 22785318 10.1038/nature11223

[42] Zhao X., Kim J., Cezar C. A., Huebsch N., Lee K., Bouhadir K., Mooney D. J., Active scaffolds for on-demand drug and cell delivery. Proc. Natl. Acad. Sci. U.S.A. 108, 67–72 (2011). 21149682 10.1073/pnas.1007862108 PMC3017202

[43] Huebsch N., Kearney C. J., Zhao X., Kim J., Cezar C. A., Suo Z., Mooney D. J., Ultrasound-triggered disruption and self-healing of reversibly cross-linked hydrogels for drug delivery and enhanced chemotherapy. Proc. Natl. Acad. Sci. U.S.A. 111, 9762–9767 (2014). 24961369 10.1073/pnas.1405469111 PMC4103344

[44] Hu W., Ishii K. S., Fan Q., Ohta A. T., Hydrogel microrobots actuated by optically generated vapour bubbles. Lab Chip 12, 3821–3826 (2012). 22899225 10.1039/c2lc40483d

[45] Wong V. W., Longaker M. T., Gurtner G. C., Soft tissue mechanotransduction in wound healing and fibrosis. Semin. Cell Dev. Biol. 23, 981–986 (2012). 23036529 10.1016/j.semcdb.2012.09.010

[46] Vermolen F. J., Javierre E., A finite-element model for healing of cutaneous wounds combining contraction, angiogenesis and closure. J. Math. Biol. 65, 967–996 (2012). 22071653 10.1007/s00285-011-0487-4

[47] Javierre E., Moreo P., Doblaré M., García-Aznar J. M., Numerical modeling of a mechano-chemical theory for wound contraction analysis. Int. J. Solids Struct. 46, 3597–3606 (2009).

[48] Benedict F. G., Miles W. R., Johnson A., The temperature of the human skin. Proc. Natl. Acad. Sci. U.S.A. 5, 218–222 (1919). 16576376 10.1073/pnas.5.6.218 PMC1091574

[49] Suarez F., Nozariasbmarz A., Vashaee D., Öztürk M. C., Designing thermoelectric generators for self-powered wearable electronics. Energ. Environ. Sci. 9, 2099–2113 (2016).

[50] Han L., Lu X., Liu K., Wang K., Fang L., Weng L.-T., Zhang H., Tang Y., Ren F., Zhao C., Sun G., Liang R., Li Z., Mussel-inspired adhesive and tough hydrogel based on nanoclay confined dopamine polymerization. ACS Nano 11, 2561–2574 (2017). 28245107 10.1021/acsnano.6b05318

[51] Ulubayram K., Cakar A. N., Korkusuz P., Ertan C., Hasirci N., EGF containing gelatin-based wound dressings. Biomaterials 22, 1345–1356 (2001). 11336307 10.1016/s0142-9612(00)00287-8

[52] Balakrishnan B., Mohanty M., Fernandez A. C., Mohanan P. V., Jayakrishnan A., Evaluation of the effect of incorporation of dibutyryl cyclic adenosine monophosphate in an in situ-forming hydrogel wound dressing based on oxidized alginate and gelatin. Biomaterials 27, 1355–1361 (2006). 16146648 10.1016/j.biomaterials.2005.08.021

[53] Zhao X., Guo B., Wu H., Liang Y., Ma P. X., Injectable antibacterial conductive nanocomposite cryogels with rapid shape recovery for noncompressible hemorrhage and wound healing. Nat. Commun. 9, 2784 (2018). 30018305 10.1038/s41467-018-04998-9 PMC6050275

[54] Xi Y., Ge J., Guo Y., Lei B., Ma P. X., Biomimetic elastomeric polypeptide-based nanofibrous matrix for overcoming multidrug-resistant bacteria and enhancing full-thickness wound healing/skin regeneration. ACS Nano 12, 10772–10784 (2018). 30481960 10.1021/acsnano.8b01152

[55] Kirker K. R., Luo Y., Nielson J. H., Shelby J., Prestwich G. D., Glycosaminoglycan hydrogel films as bio-interactive dressings for wound healing. Biomaterials 23, 3661–3671 (2002). 12109692 10.1016/s0142-9612(02)00100-x

