# Supplementary material for: Bioinspired mechanically active adhesive dressings to accelerate wound closure
Source: Sci Adv. 2019 Jul 24;5(7):eaaw3963. doi: 10.1126/sciadv.aaw3963 (PMC6656537; doi:10.1126/sciadv.aaw3963)
Supplement: http://advances.sciencemag.org/cgi/content/full/5/7/eaaw3963/DC1 [file supp_5_7_eaaw3963__index.html]

Science Advances | Science AdvancesAAASSearchScience AdvancesMenu

## Supplementary Materials

**The PDF file includes:**

- Fig. S1. Antimicrobial tests.
- Fig. S2. Histological sections on day 3 specimens.
- Fig. S3. Finite element simulation.
- Table S1. Material parameters used in finite element stimulation.
- Table S2. Comparison of mechanical and antimicrobial properties of materials related to wound care.

Download PDF

**Other Supplementary Material for this manuscript includes the following:**

- Movie S1 (.mp4 format). Finite element simulation of AAD-enabled wound contraction.

**Files in this Data Supplement:**

- Adobe PDF - aaw3963\_SM.pdf
