# Supplementary material for: Bioinspired mechanically active adhesive dressings to accelerate wound closure
Source: Sci Adv. 2019 Jul 24;5(7):eaaw3963. doi: 10.1126/sciadv.aaw3963 (PMC6656537; doi:10.1126/sciadv.aaw3963)
Supplement: Download PDF [file aaw3963_SM.pdf]

Science Advances
AAAS

advances.sciencemag.org/cgi/content/full/5/7/eaaw3963/DC1

Supplementary Materials for

Bioinspired mechanically active adhesive dressings to accelerate wound closure

S. O. Blacklow, J. Li, B. R. Freedman, M. Zeidi, C. Chen, D. J. Mooney*

*Corresponding author. Email: mooneyd@seas.harvard.edu

Published 24 July 2019, *Sci. Adv.* **5**, eaaw3963 (2019)
DOI: 10.1126/sciadv.aaw3963

The PDF file includes:

Fig. S1. Antimicrobial tests.
Fig. S2. Histological sections on day 3 specimens.
Fig. S3. Finite element simulation.
Table S1. Material parameters used in finite element stimulation.
Table S2. Comparison of mechanical and antimicrobial properties of materials related to wound care.

Other Supplementary Material for this manuscript includes the following:

(available at advances.sciencemag.org/cgi/content/full/5/7/eaaw3963/DC1)

Movie S1 (.mp4 format). Finite element simulation of AAD-enabled wound contraction.

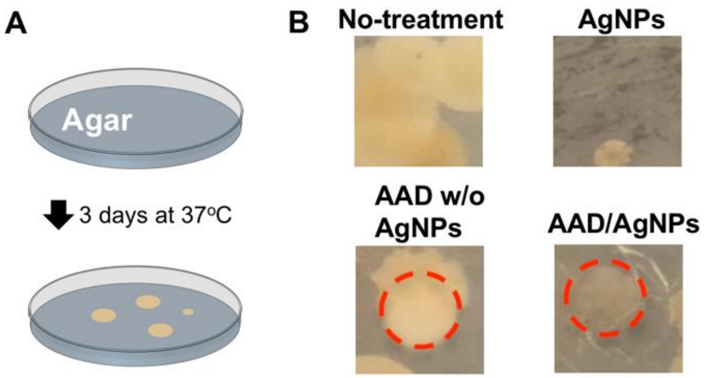

Fig. S1. Antimicrobial tests. (**A**) Schematics of the antimicrobial tests. (**B**) Digital images of the surface of agar gels with different treatments. The location of AAD without and with silver nanoparticles (AgNPs) is marked with a red dashed circle.

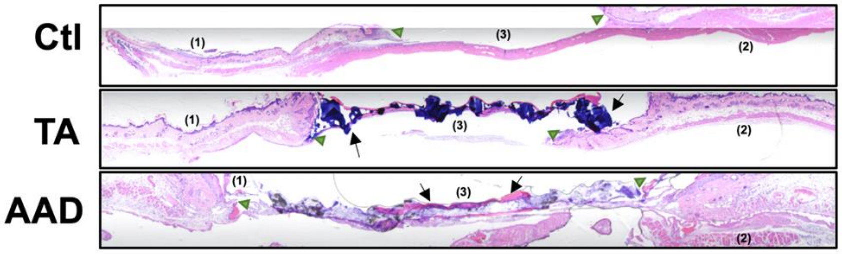

Fig. S2. Histological sections on day 3 specimens. The wound was treated with Tegaderm® as a control, tough adhesives (TA), or AAD. The wounded skin was harvested on Day 3 and H&E stained. The wound edges and the residues of adhesives are labeled with green triangles and black arrows, respectively. The epidermis, dermis, and wound bed are marked with (1), (2), and (3), respectively.

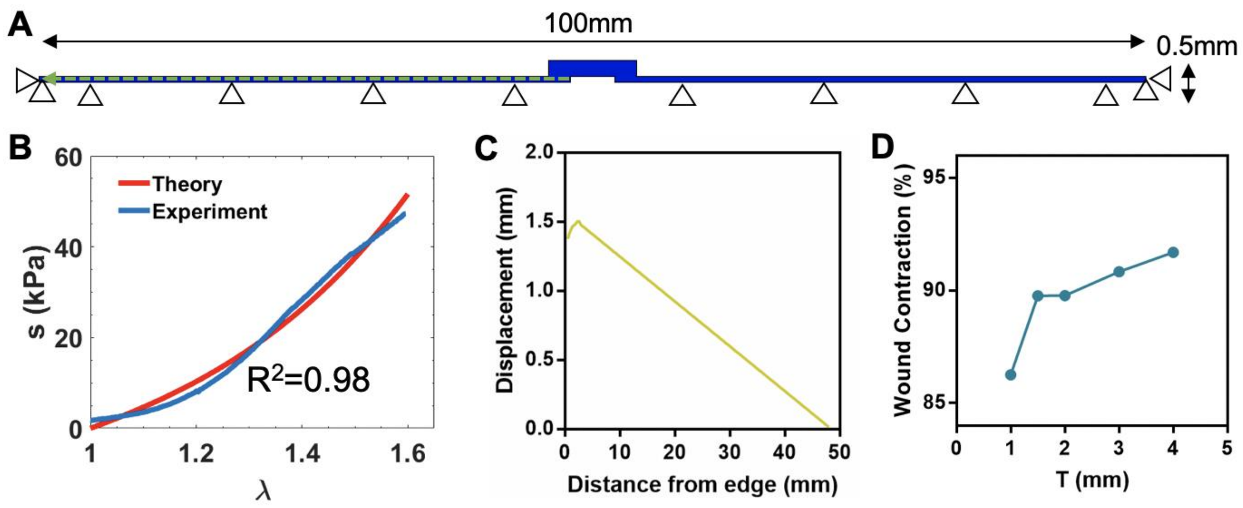

Fig. S3. Finite element simulation. (**A**) Configuration and boundary conditions of the simulation. The distance from wound edge is measured along the green dashed arrow. (**B**) Stress-strain curve of rodent skin fitted with Ogden model up to stretch ratio λ of 1.6. The nominal stress s is defined by the force divided by the initial cross-sectional area. (**C**) Horizontal displacement of the skin elements in the middle plane as function of the initial distance from the wound edge. (**D**) Wound contraction as a function of the adhesive thickness (T).

Table S1. Material parameters used in finite element stimulation. The Ogden parameters (μ, α) of rodent skin was experimentally measured and fitted in fig. S3B. The parameters of AAD, except the shear modulus (G), were cited from Cai and Suo (2014) (*19*), including cross-link density (Nv), initial swelling ratio with respect to the dry state (λ_o), initial temperature (T_o), triggered temperature (T_F), the parameters (A_o, B_o, A_1 and B_1) used to formulate the Flory-Huggins coefficient (χ) as a function of temperature (T) and the polymer fraction (ϕ) via equation, $\chi=(A_o+B_oT)+(A_1+B_1T)\,\phi$. Sample size n=5. G was calculated by one third of elastic modulus measured by tensile tests.

Rodent skin		AAD								
μ (kPa)	α	G (kPa)	Nv	λ_0	T_0 (K)	T_F (K)	A_0	B_0	A_1	B_1
25.90 (16.9-34.8)	7.08 (6.59-7.58)	**4.66**	0.01	2.36	280	310	-12	0.042	17	-0.057

Table S2. Comparison of mechanical and antimicrobial properties of materials related to wound care. The AAD and TA are compared with existing products related to wound care, including skin graft (*36*), ALGICELL® (Derma Sciences) (*17*), Band-Aid® (Johnson & Johnson), Tegaderm® (3M) (*37*,*38*), COSEAL® (*17*) and TISSEEL® (Baxter) (*39*), and Dermabond® (Ethicon) (*17*).

Products		Materials	Adhesiveness (Jm^{-2})	Toughness (Jm^{-2})	Active closure (2D strain %)	Antimicrobial
**AAD**		Alginate-PNIPAM	200	2000	64%	√
**TA**		Alginate-PAAm	1200	18000	0	×
**Wound dressings**	Skin graft[2]*	Skin	5-40	>8000	0	×
	ALGICELL®	Alginate	3	5	0	√
	Band-Aid®	Acrylate/fabric	1-10	10000	0	×
	Tegaderm®	Acrylate	20-40	2000	0	×
**Tissue adhesives**	COSEAL®	PEG	30	30	0	×
	TISSEEL®	Fibrin	60	60	0	×
	Dermabond®	Cyanoacrylate	300	2000	0	×
